# Beyond redox: vitamin C in signaling, epigenetic regulation and protein modification—relevance to cancer therapy

**DOI:** 10.1186/s13148-026-02211-z

**Published:** 2026-07-30

**Authors:** Amisha S Hebbar, Sandra Nixon, Hanumappa Ananda, Manasa Nune, Vinay Kumar Rao

**Affiliations:** 1 Manipal Institute of Regenerative Medicine, Manipal Academy of Higher Education, Manipal, India; 2 Department of Surgery, University of Connecticut School of Medicine, Farmington, CT 06030 USA

**Keywords:** Vitamin C, TETs, Histone demethylases, Epigenetics, Transcriptional regulation, Vitcylation

## Abstract

Vitamin C, a classical antioxidant, is increasingly recognized as a key regulator of cellular signaling networks, epigenetic, and post-translational regulation. As a cofactor of Fe^2+^/2-oxoglutarate-dependent dioxygenases, vitamin C promotes epigenomic changes by enhancing TET-dependent DNA demethylation and the activity of Jumonji-C domain-containing histone demethylase, thereby influencing key cellular processes including pluripotency, reprogramming, and differentiation. Recent studies have also uncovered a direct role for vitamin C in proteome regulation by modifying lysine residues, to generate vitcyl-lysine (a process termed as Vitcylation), opening new avenues for its therapeutic applications. These lesser-understood functions of vitamin C underpin its context-dependent roles in cancer and other disease states. Future mechanistic studies identifying targets of vitamin C-dependent protein modification and their consequences on epigenome regulation will be critical for advancing its therapeutic applications.

**Graphical abstract:**

**Figure:**
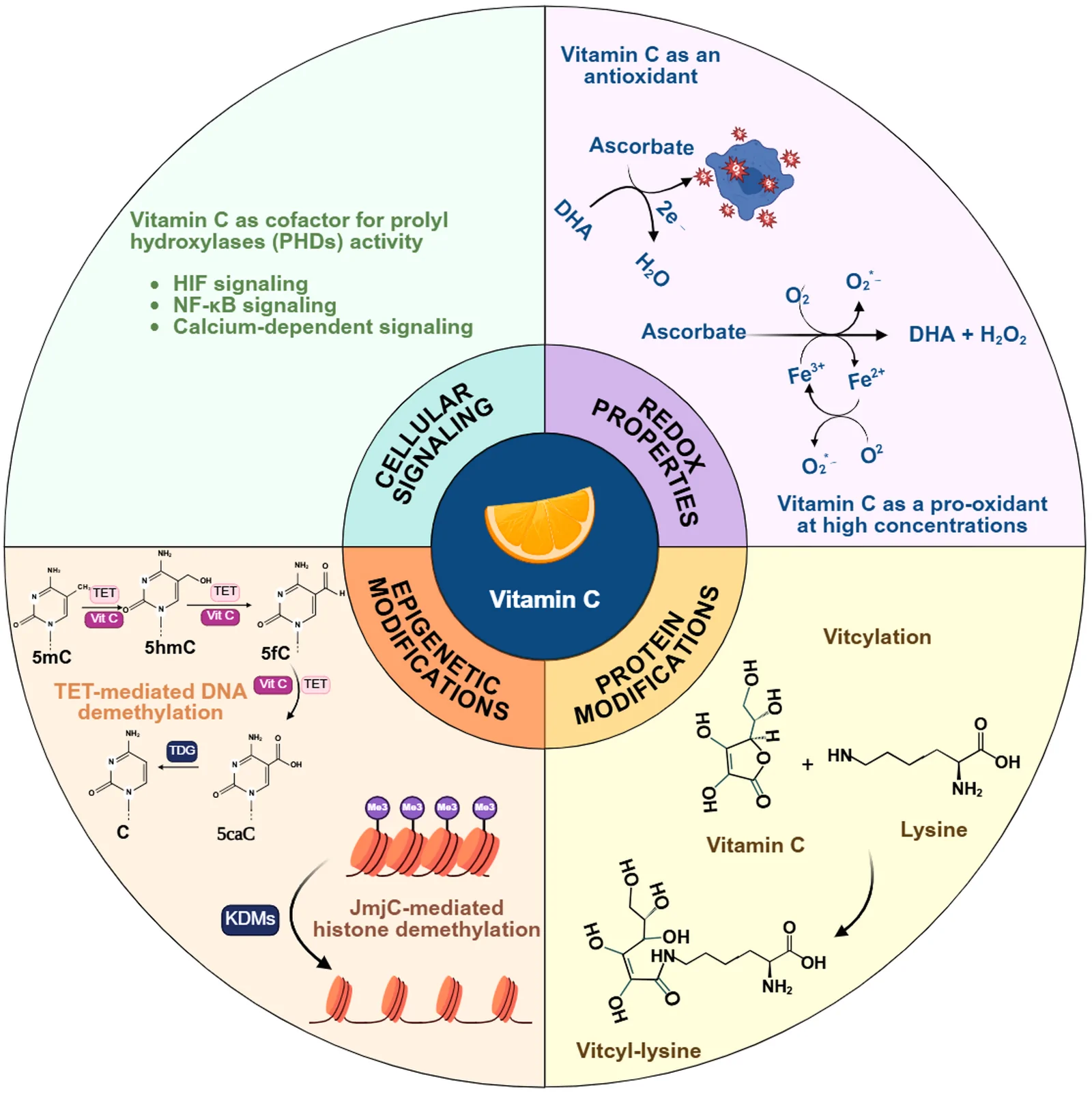

## Introduction

In mammals, vitamin C is synthesized from glucose through the uronic acid pathway, with l-gulono-γ-lactone oxidase (GULO) catalyzing the final step of ascorbic acid synthesis in the liver. However, humans cannot produce ascorbic acid because the GULO gene is non-functional. Nonetheless, the persistence of the GLUO pseudogene in humans underscores the physiological significance of vitamin C. Insufficient intake of vitamin C leads to scurvy, characterized by impaired collagen synthesis, defective wound healing, and systemic failure. Adequate vitamin C replenishment reverses the symptoms of scurvy. This is due to the activation of collagen prolyl hydroxylases, which depend on vitamin C for activity [1]. Vitamin C functions as a cofactor for a broad family of Fe^2+^ and 2-oxoglutarate-dependent dioxygenases (2OGDDs), which includes collagen prolyl and lysyl hydroxylases, dopamine β-hydroxylase, and enzymes taking part in carnitine biosynthesis [2]. Through these enzyme activities, vitamin C is linked to cellular functions, including extracellular matrix integrity, mitochondrial energy homeostasis, neurotransmitter synthesis, and vascular stability. This underscores the critical role of vitamin C in maintaining physiological balance, and hence suboptimal levels of vitamin C have been associated with increased vulnerability to tissue repair and cardiovascular diseases [3, 4].

Vitamin C functions as a cofactor for several other Fe^2+^/2OGDDs, which play a key role in DNA and histone modifications, thereby modulating key biological processes [5]. This reliance on vitamin C becomes even more evident in pathological circumstances such as cancer, aging, and inflammation, where the cellular demand for vitamin C may exceed physiological availability [6]. Moreover, recent findings provide evidence for direct protein modification by vitamin C on the lysine residue, generating vitcyl-lysine (a process termed as Vitcylation), offering significant insights into its cellular functions, including proteome regulation [7]. In this review, we summarize the role of vitamin C in regulating cellular redox homeostasis. We highlight the role of vitamin C in cell signaling and its function as a cofactor for Fe^2+^/2OGDDs in regulating the epigenome. We also emphasize its emerging roles in direct protein modification and its beneficial effects on cancer and immune regulation.

## Bioavailability, oxidative stress, and transportation of vitamin C in vivo

The bioavailability of vitamin C in humans depends on interactions among dietary intake, intestinal absorption, tissue uptake, redox cycling, and the expression of cellular transporters. While fat-soluble vitamins are passively absorbed, vitamin C exhibits saturable absorption and is actively transported, indicating that it plays a dual role as both a micronutrient and a redox-active metabolite. The concentration of vitamin C in plasma under physiological dietary conditions is 40–80 µM, while concentrations in tissues vary, indicating tissue-specific requirements and active transport processes [8]. At 30–100 mg/day of intake, the intestinal absorption exceeds 80%, while higher doses do not increase their concentrations in the plasma due to transporter saturation and renal excretion. Hence, doses above 1 g/day produce only a minor increase in plasma ascorbate levels, thereby highlighting differences in physiological and pharmacological requirements. For instance, vitamin C is accumulated by immune cells, neurons, and brain tissue at millimolar concentrations, whereas plasma levels are much lower. This indicates a high functional requirement for vitamin C in these organs and suggests roles beyond its systemic antioxidant function, reflecting its critical role in maintaining cellular redox homeostasis. Recent evidence suggests that vitamin C acts in concert with glutathione to protect cells against oxidative stress, and that impairment of glutathione biosynthesis worsens the effects of vitamin C deficiency [9]. Beyond its antioxidant function, glutathione-dependent redox regulation influences methylation-dependent cellular processes by linking oxidative stress with one-carbon metabolism and methylation capacity. Alterations in cellular redox status can affect methionine synthase activity and the availability of methyl donors, thereby modulating DNA methylation and gene expression [10]. Hence, this interplay between vitamin C and glutathione metabolism may be particularly important in cell types exposed to high levels of oxidative stress, including activated immune cells and neurons.

Notably, inflammation, trauma, and cancer are associated with lower plasma ascorbate concentrations even when dietary intake is adequate [4]. This is partly due to increased oxidative turnover as vitamin C is used up faster as an antioxidant to quench the free radical production [11]. Furthermore, the processes of inflammation and disease can alter transporter expression and increase cellular demand, as immune cells, especially T cells, require millimolar concentrations of vitamin C for maturation and function, actively drawing it from plasma [12]. As a result, plasma vitamin C concentration may not reflect tissue level sufficiency in disease contexts. Notably, intravenous administration of vitamin C circumvents intestinal saturation and increases plasma concentration more than oral administration. Hence, the route of administration may be critical for the therapeutic application of vitamin C, especially in the context of cancer.

Vitamin C protects cells as an antioxidant by scavenging free radicals and reactive oxygen species under normal physiological conditions [13]. In vivo vitamin C exists in multiple interconvertible redox states. The predominant reduced form, l-ascorbic acid (ascorbate), functions as a potent electron donor in both enzymatic and non-enzymatic reactions [14]. Upon donating a free electron, ascorbate is converted into ascorbate radical (semi-hydroascorbate), which is further oxidized to form dehydroascorbic acid (DHA), the fully oxidized form of ascorbic acid. While DHA is unstable in an aqueous environment and lacks direct antioxidant capacity, it can be recycled back to ascorbate. Also, cycling between ascorbate and its oxidized form DHA is associated with other cellular antioxidant networks, including glutathione, thioredoxin, and NADPH-dependent reductase systems, and through this coupling, vitamin C functions as both an antioxidant and also as a regulator of intracellular redox signaling [15]. Specifically, at higher concentrations in the presence of metal ions, vitamin C acts as a pro-oxidant by generating hydrogen peroxide [16]. This context-dependent redox behavior of vitamin C is a key factor in its functional versatility. In normal tissues, robust antioxidant defenses buffer potential pro-oxidant effects and maintain a redox equilibrium. In cancer cells exhibiting compromised redox buffering capacity, pharmacological vitamin C exposure renders them more vulnerable to oxidative stress [17].

The tissue distribution and cellular uptake of vitamin C are mainly driven by two transport systems that recognize different redox forms of the micronutrient: (a) Sodium-dependent vitamin C transporters (SVCT1 and SVCT2, encoded by SLC23A1 and SLC23A2, respectively), responsible for transporting reduced ascorbate, (b) facilitative glucose transporters (GLUTs), which help in the uptake of DHA [18]. SVCT1 is largely expressed in epithelial tissues, such as the intestine and kidney tubules, helping in renal vitamin C absorption and thereby maintaining systemic vitamin C homeostasis [19]. Simultaneously, SVCT2 is expressed in a wide variety of tissues, including the brain, immune cells, and endocrine organs, where it facilitates the uptake of ascorbate required for their physiological functions [20].

GLUT1, GLUT3, and GLUT4 facilitate the uptake of DHA, reflecting the structural similarity between DHA and glucose. Upon internalization, DHA is reduced back to ascorbate inside cells, enabling cells to accumulate ascorbate. In cancer, the expression of these receptors is altered, and as a result, vitamin C availability in tissues is reshaped [21]. In the cancer cells, upregulation of GLUT transporters may increase the uptake of DHA, thereby altering glucose metabolism toward vitamin C-dependent redox stress, causing therapeutic effects [17]. Hence, the coordinated function of GLUTs and SVCTs is critical for controlling vitamin C distribution across tissues, enabling context-dependent redox stress in pathological conditions.

## Vitamin C as a regulator of cellular signaling

Vitamin C predominantly functions to protect cells from oxidative damage by regulating cellular redox homeostasis [2, 22]. Vitamin C regulates transcription through oxygen-sensing pathways, notably hypoxia-inducible factor (HIF) signaling. Prolyl hydroxylase domain proteins (PHDs) are Fe^2+^ and 2-oxoglutarate-dependent dioxygenases, which utilize vitamin C for their activity. Under normoxia, PHDs and the factor inhibiting HIF (FIH) hydroxylate HIF-1α on proline and asparagine residues, leading to degradation via the proteasome pathway and transcriptional repression [23]. Vitamin C deficiency impairs HIF-1α activity even under normoxic conditions and activates a pseudo-hypoxic transcriptional program, which leads to upregulation of genes involved in angiogenesis, cell survival, and glycolysis, which are frequently exploited by cancer cells [1, 24]. Hence, by regulating HIF-1α hydroxylation, vitamin C restrains aberrant hypoxia-driven gene expression changes.

Vitamin C is also known to regulate calcium-dependent signaling in the neuronal system. Vitamin C interacts with various calcium-permeable channels and receptors, limiting the excessive accumulation of intracellular calcium that can trigger glutamate-induced neurotoxicity [25]. This regulation of calcium-dependent signaling by vitamin C helps maintain calcium homeostasis and affects neurotransmitter release and synaptic activity. Additionally, through redox potential, vitamin C influences secondary messengers such as cAMP and nitric oxide (NO) in signaling pathways, thereby integrating redox balance with signal transduction processes [26].

**Fig. 1:**
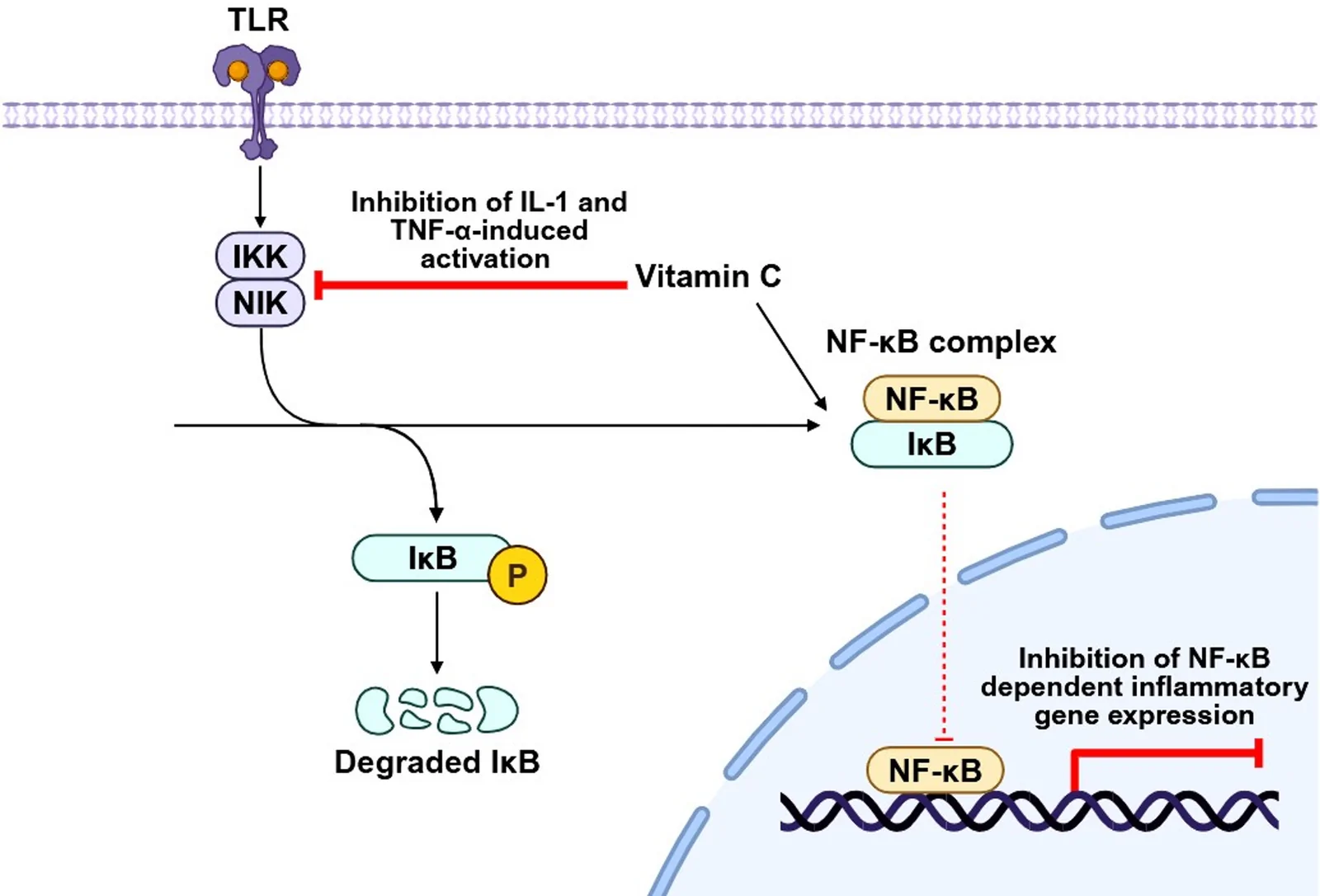
Anti-inflammatory effect of vitamin C via inhibition of NF-κB signalling. Vitamin C inhibits NF-κB activation by reducing intracellular ROS levels, stabilizing IκBα, preventing NF-κB nuclear translocation, and inhibiting the transcription of inflammatory genes. The figure was generated using Biorender.com

Vitamin C also exerts a significant anti-inflammatory effect by suppressing Nuclear Factor Kappa-light-chain-enhancer of activated B cells (NF-κB) signaling through multiple complementary mechanisms. One such mechanism involves inhibition of IκBα phosphorylation and degradation induced by pro-inflammatory cytokines such as IL-1 and TNF-α, thereby preventing NF-κB nuclear translocation and transcriptional activation [27]. Under pro-inflammatory stimulation, cytokines activate NF-κB via a kinase cascade involving NF-κB-inducing kinase (NIK) and the IκB kinase (IKK) complex, which phosphorylates IκBα, leading to its degradation and subsequent nuclear translocation of NF-κB. Vitamin C inhibits this pathway by suppressing NF-κB signaling through dual mechanisms: redox-dependent and direct enzymatic regulation. It inhibits TNF-α-induced activation of the kinases (NIK and IKK) by reducing the intracellular ROS (Fig. 1).

On the other hand, the oxidized form of vitamin C, DHA, directly inhibits IKK activity, preventing IκBα phosphorylation and degradation [28]. In addition, vitamin C is known to activate the p38 MAPK pathway, which contributes to the stabilization of IκBα and the suppression of NF-κB-dependent inflammatory gene expression [29].

Vitamin C regulates metabolic genes linked to glycolysis and lipid metabolism, thereby coupling metabolic status to gene expression responses [30]. Mechanistically, it promotes DNA demethylation while enhancing prolyl hydroxylase activity to drive HIF-1α degradation and suppress glycolytic gene expression, including GLUT1, LDHA, and PDK1. At higher concentrations, vitamin C induces redox-mediated metabolic stress, disrupting glycolytic flux and cellular energy balance [31]. Furthermore, by limiting the availability of citrate and acetyl-CoA, it inhibits lipid biosynthesis and modulates histone acetylation, thereby linking metabolic flux to chromatin and transcriptional regulation.

## Vitamin C as a regulator of the epigenome

Vitamin C has emerged as a critical regulator of transcription and epigenetic state of cells. This is primarily due to its function as a cofactor for Fe^2+^/2OGDDs, including Ten-eleven translocation (Tet) family proteins, Jumonji C (JmjC) domain-containing histone demethylases, and DNA and RNA demethylases of the AlkB homolog (ALKBH) family [32–34].

Vitamin C helps maintain iron in its reduced state (Fe^2+^), thereby enhancing the catalytic activity of these enzymes [35]. Tet proteins (Tet1, Tet2, and Tet3) catalyze sequential oxidation of DNA 5-methylcytosine (5mc) to 5-hydroxymethylcytosine (5hmc), 5-formylcytosine (5fc), and 5-carboxylcytosine (5cac), initiating active DNA demethylation [36, 37]. Ascorbate availability is a rate-limiting factor for Tet-mediated DNA demethylation, particularly pronounced in cells with lower vitamin C levels.

Similarly, JmjC domain histone demethylases (KDMs) remove methylation on histones, including repressive H3K9me2 and H3K27me3 marks [38]. Through both DNA and histone demethylation, vitamin C exerts profound effects on cell fate decisions. A recent study shows that a deficiency of maternal vitamin C can disrupt DNA methylation homeostasis, thereby contributing to abnormalities, especially in the presence of risk factors that affect epigenetic regulation [39].

**Fig. 2:**
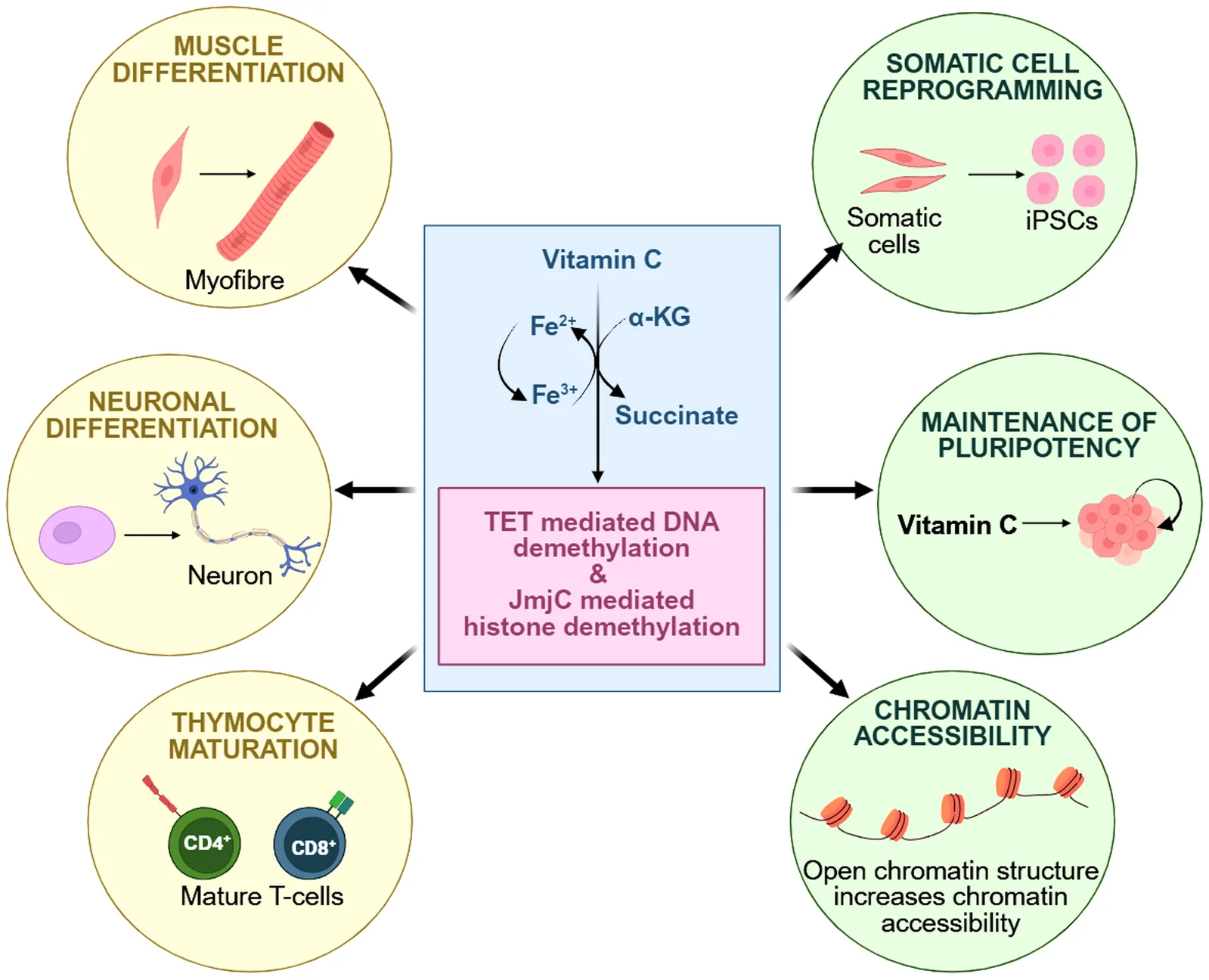
Role of Vitamin C in stem cell reprogramming and differentiation. Vitamin C acts as a cofactor for TETs and histone demethylases, leading to DNA and histone demethylation and thereby regulating gene expression. The figure was generated using Biorender.com

Vitamin C enhances stem cell reprogramming and maintains pluripotency by establishing an open chromatin configuration in stem cells [5, 40]. Vitamin C supports embryonic stem cell (ESC) proliferation and enhances somatic cell reprogramming into induced pluripotent stem cells (iPSCs) by stimulating Jumonji C domain-containing histone demethylases (JHDMs). Vitamin C enhances the activity of JHDM1a/1b, thereby demethylating H3K36me2/3 in mouse embryonic fibroblasts and promotes reprogramming of fibroblasts. Consistently, overexpression of JHDM1a/1b enhanced reprogramming, whereas its knockdown impaired it. Vitamin C-mediated JHDM1b activation induces the expression of microRNAs, which increases reprogramming efficiency [41]. Vitamin C, through activation of JHDM1a/1b, promotes the reduction of H3K9me2 in embryonic stem cells and helps in efficient reprogramming to induced pluripotent cells (iPSCs) [42, 43] (Fig. 2).

Dysregulation of KDM activity influences genomic stability in both stem cells and cancer cells. For example, KDM5B enhances double-stranded break repair by recruiting factors Ku70 and BRCA1 in osteosarcoma cells, whereas KDM5 deficiency promotes spontaneous DNA damage and p53 activation [44]. However, aberrant overexpression of certain KDMs, such as KDM2B or KDM4A, can impair homologous recombination repair, promoting genomic instability [45, 46]. Hence, vitamin C-mediated modulation of KDM activity might either preserve genome integrity or alter DNA repair capacity, in a context-dependent manner. While these studies do not directly explore vitamin C, they focus on the broader functional importance of KDMs, indicating that vitamin C-mediated regulation of KDM activity may influence genome stability and DNA repair capacity in a context-dependent manner.

Similarly, vitamin C enhances iPSC reprogramming by activating Tet enzymes. Vitamin C augments Tet activity and increases 5hmc levels in ESCs and during fibroblast reprogramming to iPSCs, and such effects of vitamin C are lost in Tet-depleted cells [40, 47]. Tet protein depletion in fibroblasts hinders iPSC generation, while its ectopic expression enhances the generation of iPSCs [48, 49]. These studies indicate that vitamin C functions in DNA demethylation through Tet proteins (Fig. 2).

Given that Tet proteins, particularly Tet2, function as tumor suppressors in the hematopoietic system and are frequently mutated in myelodysplastic syndrome (MDS) and acute myeloid leukemia (AML), vitamin C-mediated regulation of Tet activity has important implications for hematopoietic stem cell (HSC) fate [50]. Genetic studies have also shown that loss of Tet proteins disrupts lineage commitment and promotes leukemia induction by reducing 5hmc and altering gene expression [51, 52].

In addition to enhancing stem cell reprogramming, vitamin C plays a significant role in early T-cell development by promoting key stages of thymocyte maturation. In both in vitro and in vivo, ascorbic acid was shown to accelerate the transition of immature thymic progenitors from double negative (CD4− and CD8−) stages to double positive (CD4+ and CD8+) stages in a dose-dependent manner and is independent of T-cell receptor (TCR) gene rearrangement processes [12]. Additionally, vitamin C enhances the efficiency of TCR-mediated signaling and the development of CD8 single-positive cells (Fig. 2). When combined with inhibitors of histone lysine methyltransferases (KMTs) or DNA methyltransferases (DNMTs), using BIX01294 and RG108, which are specific for H3K9 methyltransferases KMT1C/D and all DNMTs, respectively, it further improved thymocyte progression, highlighting its role in modulating signaling and epigenetic mechanisms to drive T-cell lineage commitment and selection [53].

Vitamin C also plays a role in muscle differentiation (Fig. 2). It promotes skeletal muscle differentiation through a mechanism involving epigenetic regulation, transcriptional activation, and signaling modulation, where vitamin C enhances DNA and histone demethylation that leads to activation of myogenic genes—MyoD and Myogenin. Parallelly, it directly regulates myogenic transcription factors and promotes myotube formation through interactions with proteins such as CSRP3 [54, 55]. Furthermore, vitamin C mechanistically drives the demethylation of DNA and histones at promoters of neurogenic genes through TETs and Jumonji-domain demethylases. This results in the activation of transcriptional programs required for neuronal lineage commitment, including upregulation of the fundamental genes involved in neuronal differentiation, such as the dopaminergic lineage markers Foxa2, Lmx1α, and Nurr1 [56]. Dopamine β-hydroxylase (DBH) is a well-known enzyme that converts dopamine to norepinephrine, and vitamin C serves as an essential cofactor for DBH, maintaining its catalytic activity by donating the electrons required for catecholamine biosynthesis [57, 58]. Thus, vitamin C contributes to both the establishment of dopaminergic neuron identity and the synthesis of neurotransmitters required for neuronal function. In fetal mid-brain neural stem cells, the expression of key dopaminergic markers, including tyrosine hydroxylase (TH), vesicular monoamine transporter 2 (VMAT2), and dopamine transporter (DAT), is enhanced by vitamin C through TET1 and JMJD3-mediated epigenetic regulation. Collectively, this evidence demonstrates that vitamin C supports both functional maturation and differentiation of dopaminergic neurons by coordinating neurotransmitter production with epigenetic regulation [59].

## Vitamin C’s role in direct protein modifications

Under low pH conditions, the oxidized form of vitamin C, DHA, undergoes further oxidation to form diketogulonate (DKG), which leads to protein modifications called ascorbylation in plants and food. However, under physiological conditions, post-translational modifications (PTMs) of proteins, such as acetylation, phosphorylation, methylation, ubiquitination, and SUMOylation, modify specific amino acids, thereby affecting their functions. Interestingly, emerging evidence indicates that vitamin C, under physiological conditions, directly modifies proteins post-translationally to produce vitcyl-lysine, a process termed “vitcylation”, distinct from ascorbylation [7].

Vitcylation occurs over a broader pH range, with a peak at pH 9–10 and involves chemical modification of peptides and proteins, positioning vitamin C as a direct substrate. Vitcylation involves the attachment of ascorbate-derived moiety to the *ε-*amino group of lysine residues, forming stable amide- or imine-like linkages depending on the redox environment. Vitcylation of proteins depends on the dose, pH, and amino acid sequence in both cell-free and living cells. Vitcylation has been shown to occur specifically at the STAT1 K298 residue, where it interferes with STAT1 dephosphorylation, thereby sustaining STAT1 in a phosphorylated and transcriptionally active state. Consequently, this modification of STAT1 enhances the expression of interferon-stimulated genes (ISGs), increases major histocompatibility complex (MHC) and human leukocyte antigen (HLA) class 1 expression, and biases the immune response towards an anti-tumor phenotype within the tumor microenvironment. Proteomic analysis reveals vitcylation of a wide range of proteins involved in metabolism, cellular stress response, and immune modulation, indicating that vitcylation targets multiple protein classes. Vitcylation alters lysine charge and steric properties, potentially affecting its protein–protein interactions and cellular localization [7]. Although the study identified vitcylation of several proteins and peptides, the functional significance of these modifications across physiological and disease contexts remains largely unexplored. Furthermore, the physiological conditions that regulate vitcylation, itspotential reversibility, molecular basis of target selectivity, and its crosstalk with other post-translational modifications remain important open questions for future investigation.

## Expanding role in cancer therapies

Linus Pauling and Ewan Cameron in the 1970s proposed that high doses of vitamin C could improve the quality of life and survival of cancer patients [60]. These early studies were controversial due to methodological limitations and variable outcomes across studies. However, since then, vitamin C has generated significant interest as a promising anticancer agent, in a context-dependent manner, as it functions both as an antioxidant and a pro-oxidant. Vitamin C acts as an antioxidant at physiological concentrations by scavenging ROS and limiting DNA damage. However, at pharmacological concentrations, attained through intravenous administration, it exhibits pro-oxidant activity by generating hydrogen peroxide through metal-catalyzed oxidation and thereby promoting cell death [61]. Furthermore, when vitamin C is administered in high doses, it gets oxidized to the ascorbate radical, which reduces iron to the ferrous form. Later, in the Fenton reaction, ferrous iron reacts with oxygen to produce the superoxide anion (O_2_^−^), which can cause toxic levels of hydrogen peroxide (H_2_O_2_). As a result, cancer cells experience elevated basal oxidative stress, deregulated iron metabolism, and disrupted redox buffering systems, leading to cell death. However, in contrast, normal cells detoxify hydrogen peroxide by higher catalase and glutathione peroxidase activity, which confer resistance to oxidative damage [62]. Hence, in cancer cells, a pharmacological dose of vitamin C, catalase deficiency, and increased ROS levels render them vulnerable to its pro-oxidant effects.

In addition, vitamin C disrupts cancer cell metabolism by targeting glycolysis and redox homeostasis. DHA enters cancer cells through GLUT transporters and is rapidly converted to ascorbate at the expense of intracellular glutathione and NADPH, causing oxidative stress. This eventually leads to a decrease in the glycolytic enzyme activity and decreased production of ATP in cancer cells [21, 63]. Vitamin C also regulates NRF2 signaling by altering the intracellular redox status. At pharmacological concentrations, the pro-oxidant activity of vitamin C triggers a transient oxidative stress, activating NRF2 and inducing antioxidant response genes. Parallelly, vitamin C also influences MAPK signaling in a context-dependent manner by suppressing ERK-mediated pro-survival pathways while activating stress-responsive kinases such as p38 MAPK, thereby contributing to reduced tumor cell viability and enhanced stress response [64].

Vitamin C also supports the expression of tumor suppressor genes through DNA demethylation, thereby reducing tumor aggressiveness and enhancing the efficacy of anti-tumor therapies. For example, vitamin C restores the expression of tumor suppressor genes such as CDKN2A (p16) by promoting Tet-dependent DNA demethylation, thereby reversing aberrant hypermethylation and inhibiting cancer cell proliferation [65]. Furthermore, the study by Agathocleous et al. [6] showed that vitamin C enhances Tet2 activity, promoting DNA demethylation and reactivation of tumor suppressor pathways. Thereby suppressing leukemogenesis and restoring normal hematopoietic differentiation.

A high dose of vitamin C is known to exhibit selective cytotoxicity against tumor cells in vitro, with its anticancer activity strongly concentration dependent. Evidence indicates that when plasma concentrations exceed 1 nmol/L, vitamin C can strongly trigger tumor cell necrosis [2]. Additionally, vitamin C has been reported to suppress the Warburg effect, thereby reducing cancer cell proliferation by interfering with glucose-driven metabolic reprogramming. This metabolic disruption is known to preferentially target malignant cells while causing limited toxicity to normal, healthy cells [66].

**Fig. 3:**
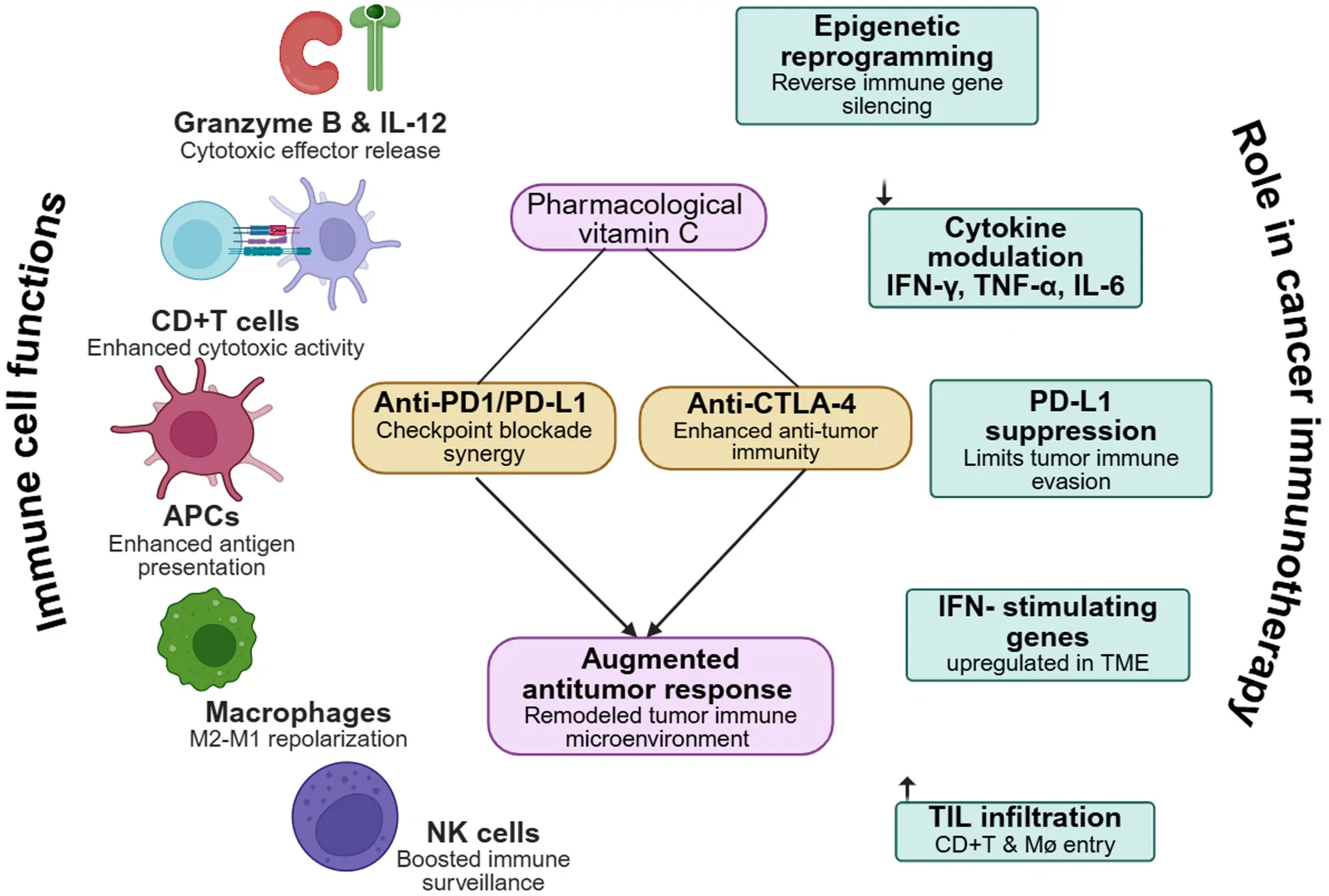
Role of vitamin C in cancer immunotherapy as an anti-cancer agent. Vitamin C promotes immune cell function through epigenetic reprogramming and supports anti-tumor immunity. Additionally, at high concentrations, it induces oxidative stress in cancer cells, leading to selective cytotoxicity. The figure was generated using Biorender.com

Beyond these cytotoxic and metabolic effects, vitamin C plays an important role in shaping anti-tumor immunity by modulating both innate and adaptive immune responses. It markedly enhances the activity of cytotoxic CD8+ T cells, Natural Killer (NK) cells, and antigen-presenting cells, thereby supporting tumor immune surveillance [67]. Vitamin C promotes immune cell differentiation and functional reprogramming by coordinating epigenetic pathways, redox balance, and intracellular signaling networks [68]. It alleviates immunosuppressive signaling and enhances antigen presentation in the tumor microenvironment. Notably, vitamin C enhances tumor-infiltrating lymphocytes and reverses epigenetic silence of immune-related genes, thereby augmenting the efficacy of immune checkpoint blockade [69, 70]. At the cytokine level, vitamin C substantially reduces the production of pro-inflammatory mediators, such as IFN-γ, TNF-α, and IL-6 [71, 72]. These are known to induce PD-L1 expression in tumor cells under inflammatory conditions, suggesting its role in limiting immune invasion [73]. Importantly, pharmacological doses of ascorbic acid treatment enhance the ability of lymphoma cells to provoke an immune response, facilitate the infiltration of CD8+ T cells and macrophages into tumors, and promote the release of granzyme B and IL-12, thereby synergizing with anti-PD1 checkpoint inhibitors [69] (Fig. 3).

Integration of vitamin C with immune checkpoint inhibitors targeting cytotoxic T-lymphocyte-associated protein 4 (CTLA-4) and the programmed cell death-1/programmed cell death ligand-1 (PD-1/PDL-1) axis has shown promising effects in enhancing anti-tumor immunity. A high dose of vitamin C is known to remodel the tumor immune microenvironment by increasing CD8+ T-cell infiltration and cytotoxic effector function, partly through enhanced expression of interferon-stimulated genes [74]. It also influences macrophage polarization, promoting a shift from immunosuppressive M2-like phenotypes towards pro-inflammatory M1-like states, thereby reducing tumor-associated immune suppression [75] (Fig. 3).

## Recent clinical trials involving vitamin C (2012–2025)

Recent discoveries on the role of vitamin C in cellular physiology have sparked interest in high-dose intravenous vitamin C treatment in clinical trials. Since 2012, many studies have been registered on ClinicalTrials.gov that have evaluated the efficacy of intravenous administration of vitamin C either as monotherapy or as an adjuvant to cancer therapies like chemotherapy, radiation therapy, and immunotherapy across different types of cancers, including pancreatic, ovarian, lung, and colorectal cancers [76, 77]. Phase I and II studies have constantly demonstrated that intravenous vitamin C is relatively safe and well-tolerated with minimal adverse effects and reported to improve the quality of life in cancer patients [78]. Regardless of mixed outcomes from various studies, accumulating evidence indicates improved therapeutic effects when vitamin C is administered with standard treatment regimens rather than alone [79] (Table 1).

**Table 1:** Clinical trials (2006–2026) involving vitamin C as an anticancer agent

Trial ID	Year	Cancer type(s)	Treatment used	Phase	Status	Key outcome	Reference
NCT00329498	2006	Advanced cancers (breast, prostate, colorectal, lung, others prostate, colorectal, lung)	IV vitamin C monotherapy	Phase I	Completed	High-dose IV vitamin C was well tolerated with minimal toxicity, but no objective tumor responses were observed; concluded limited efficacy as monotherapy	https://clinicaltrials.gov/study/NCT00329498
NCT00441207	2007	Advanced cancers	IV vitamin C monotherapy	Phase I	Completed	Early study demonstrating safety and feasibility of IV vitamin C; no objective tumor regression observed	https://clinicaltrials.gov/study/NCT00441207
NCT01080352	2010	Advanced solid tumors	IV vitamin C	Phase I	Completed	Established that pharmacologic ascorbate achieves millimolar plasma concentrations safely, with no significant anti-tumor responses observed in advanced cancer patients	https://clinicaltrials.gov/study/NCT01080352
NCT01050621	2010	Advanced solid tumors	IV vitamin C	Phase I	Completed	Confirmed safety and pharmacokinetics of IV vitamin C; no meaningful anti-tumor activity observed as a single agent	https://clinicaltrials.gov/study/NCT01050621
NCT02248584	2014	Pancreatic cancer	IV vitamin C + Gemcitabine	Phase I/II	Completed	Combination of IV vitamin C with gemcitabine showed good tolerability and suggested improved tumor response and survival trends compared to historical controls but not statistically powered for efficacy	https://clinicaltrials.gov/study/NCT02248584
NCT02969681	2016	Pancreatic cancer	IV vitamin C + Chemotherapy	Phase II	Completed	Reported improved progression-free survival (PFS) and overall survival (OS) trends in pancreatic cancer patients receiving combination therapy; treatment was well tolerated with manageable toxicity	https://clinicaltrials.gov/study/NCT02969681
NCT03146962	2017	Advanced solid tumors (including pancreatic, colorectal, lung)	IV vitamin C + Chemotherapy	Phase I	Completed	Demonstrated that high-dose IV vitamin C in combination with chemotherapy is safe and tolerable, with no dose-limiting toxicities reported: limited evidence of disease stabilization in a subset of patients, but no definitive efficacy conclusions	https://clinicaltrials.gov/study/NCT03146962
NCT04033107	2019	Metastatic colorectal cancer (KRAS-mutant)	IV vitamin C + Chemotherapy	Phase II	Ongoing	Ongoing: no results reported yet. Hypothesis-driven trial targeting KRAS-mutant colorectal cancer metabolic vulnerability; outcomes pending	https://clinicaltrials.gov/study/NCT04033107
NCT04046094	2019	Bladder cancer (urothelial carcinoma)	IV vitamin C + Immunotherapy	Phase I/II	Recruiting	Ongoing: no results yet. Evaluating whether vitamin C enhances immune checkpoint inhibitor response in bladder cancer	https://clinicaltrials.gov/study/NCT04046094
NCT04463459	2020	Pancreatic ductal adenocarcinoma	IV vitamin C + Chemotherapy	Phase II	Ongoing	Ongoing: no published results yet. Designed to assess overall survival (OS), progression-free survival (PFS), and response rates in pancreatic cancer	https://clinicaltrials.gov/study/NCT04463459
NCT04801511	2021	Locally advanced rectal cancer	High-dose IV Vitamin C (± standard therapy)	Phase I	Recruiting	Recruiting: no results available. Primary aim is to determine maximum tolerated dose (MTD) and pharmacokinetics in rectal cancer patients undergoing neoadjuvant therapy	https://clinicaltrials.gov/study/NCT04801511
NCT05932511	2023	Advanced solid tumors	IV vitamin C + Combination therapy	Phase I	Recruiting	Recruiting: no results available. Focused on dose escalation and biomarker-based response evaluation	https://clinicaltrials.gov/study/NCT05932511
NCT06018896	2023	Advanced solid tumors	IV vitamin C + Targeted therapy	Phase I	Recruiting	Recruiting: no results available. Focused on evaluating combination with targeted therapies in a precision oncology framework	https://clinicaltrials.gov/study/NCT06018896
NCT03682029	2024	Myelodysplastic syndromes, chronic myelomonocytic leukemia-1, cytopenia	Dietary supplement of vitamin C	Phase II	Ongoing	Ongoing: no results available. The study aims to evaluate if oral vitamin C may change the biology of low-risk myeloid malignancies by reversing the epigenetic changes characteristic of these disease entities	https://clinicaltrials.gov/study/NCT03682029
NCT07121036	2025	Malignant solid tumors	Hogh dose vitamin C	Phase I	Recruiting	Recruiting: no results available. Main aim is to evaluate high dose vitamin C infusion regimen based on pharmacokinetic characteristics for patients with advanced malignant solid tumors	https://clinicaltrials.gov/study/NCT07121036
NCT07310407	2026	Metabolic dysfunction-associated steatotic liver disease (MASLD)	Vitamin C as monotherapy versus vitamin C + Rutin	Phase II	Not yet recruiting	Not yet recruiting: no results available. The study aims to evaluate the combined effects of Rutin with vitamin C versus vitamin C alone on selected oxidative stress markers, inflammation, hepatic steatosis regression, and associated metabolic parameters in patients with MASLD	https://clinicaltrials.gov/study/NCT07310407
NCT07476443	2026	lung, bladder, ovarian, head and neck cancers	Vitamin C + Coenzyme q10 + Cisplatin	Phase II and Phase III	Not yet recruiting	Not yet recruiting: no results available. The study aims to evaluate the protective effects of vitamin C and coenzyme q10 against cisplatin-induced nephrotoxicity in the chemotherapy-naive cancer patients	https://clinicaltrials.gov/study/NCT07476443

A major challenge in the clinical translation of vitamin C is the gap between the preclinical outcomes and comparatively inconsistent findings observed in clinical trials. While differences in route of administration and achievable plasma concentrations have often been cited to be the main reasons, accumulating evidence suggests that the disparity is much more complex [74, 80]. Tumor-specific factors, including redox buffering capacity, metabolic heterogeneity, and the composition of the tumor microenvironment, may influence responsiveness to vitamin C, thereby contributing to variability in therapeutic outcomes. Furthermore, depending on the concentration, cellular environment, and disease state, vitamin C may function as a redox modulator, epigenetic regulator, immune modulator, or metabolic stressor, making it difficult to explain its universal mechanism of action across all cancer types [81]. The therapeutic responses observed in experimental models may not be reproducible in patient populations characterized by greater biological complexity and inter-individual variability, thereby affecting vitamin C uptake, distribution, and mechanism of action. Furthermore, intra-tumoral differences in iron availability, hypoxic status, and antioxidant enzyme activity can alter H_2_O_2_ generation and detoxification, thereby influencing sensitivity to pharmacological ascorbate [17, 80].

Another major challenge is the lack of reliable biomarkers for patient selection, and emerging evidence suggests that responses to vitamin C may depend on tumor genotype, transporter expression, epigenetic status, and redox metabolism [82–84]. Furthermore, recent clinical trials have begun to incorporate molecular stratification, underscoring the need for biomarker-guided approaches to identify patient populations most likely to benefit from vitamin C-based therapies (Table 2).

**Table 2:** Predictive biomarkers of response to vitamin C therapy and associated studies/clinical trials

Category	Biomarker	Molecular basis of Vitamin C sensitivity	Cancer types	References	Clinical trial ID
Genetic Biomarkers	KRAS/BRAF mutations	Increased GLUT1-mediated DHA uptake causes oxidative stress and GAPDH inhibition	Colorectal cancer Pancreatic cancer	[21, 85, 86]	NCT0341803 (pancreatic cancer)
	Tet2 mutation or loss of function/lower 5-hydroxymethylation (5hmc)	Enhanced Tet activity restores DNA demethylation and differentiation programs	Myeloid malignancies/hematological neoplasia	[87–90]	NCT03682029
	IDH1/IDH2 mutations	Mutant IDH suppresses Tet function through 2-HG. Vitamin C restores Tet-dependent epigenetic regulation	Myeloid leukemia	[91]	No dedicated biomarker-driven trial yet
Vitamin C Transport Biomarkers	GLUT1 high expression	Elevated GLUT1 facilitates DHA uptake and intracellular ROS accumulation	KRAS/BRAF-driven tumors	[21]	NCT03418038
	SVCT1/SVCT2	SVCT2 regulates intracellular vitamin C accumulation and bioavailability	Solid tumors, hematologic malignancies	[92]	No dedicated biomarker-driven trial yet
Hypoxia-related biomarkers	HIF1-α activation	Vitamin C acts as a cofactor for prolyl hydroxylases, regulating HIF1 degradation	Hypoxic solid tumors	[93]	No dedicated vitamin C-based trial yet
Differential gene expression	SERPINE1 and SERP1NB7	Pharmacological vitamin C exposure downregulates the expression of both genes	Non-small cell lung cancer	[94]	No registered clinical trial yet

## Future perspectives

Vitamin C, an essential micronutrient and an active biomolecule, has generated significant interest in medicine. It can also influence multiple processes, such as redox regulation, epigenomic regulation, and modulation of immune responses. Hence, vitamin C is unique compared to conventional drugs that target a single molecule or pathway [2, 3]. A major future direction is the advancement of context-dependent therapeutic strategies that exploit the dual redox properties of vitamin C. Vitamin C is also well positioned to serve as an adjuvant to chemotherapy and immunotherapy, thereby enhancing its efficacy and reducing toxicity [99, 100]. Additionally, vitamin C’s clinical use is supported by its safety profile and its ease of achieving pharmacological concentrations in plasma. Accumulating evidence on the role of vitamin C in regulating transcription and the epigenome adds new insights to its existing functions. Its critical role in DNA and histone demethylation, driven by Tet and Jumonji proteins, can likely be exploited for targeted epigenetic reprogramming. For instance, restoring tumor suppressor genes in cancers could be highly favorable through targeted reprogramming [6, 88]. Furthermore, the remarkable discovery of protein modifications mediated through vitamin C, vitcylation, has sparked further interest in its direct function in regulating the cellular proteome [7].

Despite these strengths, a few limitations deter its extensive use in clinical settings. Oral bioavailability of vitamin C is tightly controlled, and plasma concentrations plateau at lower levels due to limitations in the intestinal absorption and renal excretion. Also, the pro- and antioxidant effects of vitamin C, which are context-dependent, pose a double-edged sword that involves careful therapeutic evaluation [101]. Additionally, important challenges remain underexplored, including identifying the molecular features that best predict vitamin C responsiveness and selecting pharmacodynamic biomarkers to better understand therapeutic efficacy, and determining whether different cancer types require different dosing strategies. Addressing these gaps will be crucial for determining whether local vitamin C delivery could achieve therapeutic benefits beyond those achievable through conventional supplementation approaches. A comprehensive understanding of its tissue-specific dynamics and its biological integration with system-level approaches spanning metabolism, epigenetics, and immunology will be crucial for its therapeutic applications, especially in cancer.

## Data Availability

No datasets were generated or analysed during the current study.

## Abbreviations

HIF: Hypoxia inducible factor
GULO: L-gulono-γ-lactone oxidase
2OGDDs: 2-Oxoglutarate-dependent dioxygenases
DHA: Dehydroascorbic acid
GLUT1: Glucose transporter 1
GLUT3: Glucose transporter 3
GLUT4: Glucose transporter 4
SVCT1: Sodium-dependent vitamin C transporter-1
SVCT2: Sodium-dependent vitamin C transporter-2
SLC23A1: Solute carrier family 23, member 1
SLC23A2: Solute carrier family 23, member 2
PHDs: Prolyl hydroxylase domain proteins
FIH: Factors inhibiting HIF
cAMP: Cyclic adenosine monophosphate
IL-1: Interleukine-1
IL-6: Interleukine-6
TNF-α: Tumor necrosis factor-alpha
NF-κB: Nucleic factor kappa B
NIK: NF-κB-inducing kinase
IKK: Inhibitor of kappa B kinase
p38 MAPK: P38 mitogen-activated protein kinase
IκBα: Inhibitor of kappa B alpha
ROS: Reactive oxygen species
LDHA: Lactate dehydrogenase A
PDK1: 3-Phosphoinositide dependent protein kinase-1
Acetyl-CoA: Acetyl coenzyme A
ALKBH: Alkylated DNA repair protein homolog
5mc: 5-Methylcytosine
5hmc: 5-Hydroxymethylcytosine
5fc: 5-Formylcytosine
5cac: 5-Carboxylcytosine
TETs: Ten-eleven translocases
JmjC: Jumonji C domain
KDMs: Lysine demethylases
JHDMs: Jumonji C domain-containing histone demethylases
ESC: Embryonic stem cell
iPSC: Induced pluripotent stem cell
HSC: Hematopoietic stem cell
MDS: Myelodysplastic syndrome
AML: Acute myeloid leukemia
CMML: Chronic myelomonocytic leukemia
CHIP: Clonal hematopoiesis of intermediate potential
TCR: T-cell receptor
KMT: Histone lysine methyltransferase
DNMT: DNA methyltransferase
PTM: Post-translational modification
ISGs: Interferon-stimulated genes
NK: Natural killer cells
CTLA-4: Cytotoxic T-lymphocyte-associated protein-4
PD-1: Programmed cell death-1
PDL-1: Programmed cell death ligand-1
NRF2: Nuclear factor erythroid 2-related factor 2
ATP: Adenosine triphosphate
NADPH: Nicotinamide adenine dinucleotide phosphate
Foxa2: Forkhead box protein A2
Lmx1α: LIM homeobox transcription factor alpha
Nurr1: Nuclear receptor-related 1 protein
STAT1: Signal transducer and activator of transcription 1
CSRP3: Cysteine and Glycine-rich protein 3
ERK: Extracellular signal-regulatory kinase
CDKN2A: Cyclin dependent kinase inhibitor 2A
DNA: Deoxyribonucleic acid
p53: Tumor protein p53
IFN-γ: Interferon gamma
DBH: Dopamine β-hydroxylase
TH: Tyrosine hydroxylase
VMAT2: Vesicular monoamine transporter 2
DAT: Dopamine transporter
